# An Online Training Intervention on Prehospital Stroke Codes in Catalonia to Improve the Knowledge, Pre-Notification Compliance and Time Performance of Emergency Medical Services Professionals

**DOI:** 10.3390/ijerph17176183

**Published:** 2020-08-26

**Authors:** Montse Gorchs-Molist, Silvia Solà-Muñoz, Iago Enjo-Perez, Marisol Querol-Gil, David Carrera-Giraldo, Jose María Nicolàs-Arfelis, Francesc Xavier Jiménez-Fàbrega, Natalia Pérez de la Ossa

**Affiliations:** 1 Catalonian Emergency Medical System, 08908 L’Hospitalet de Llobregat, Spain; silviasola@gencat.cat (S.S.-M.); marisol.querol@gmail.com (M.Q.-G.); francescxavierjimenez@gencat.cat (F.X.J.-F.); 2 School of Medicine and Healthcare Sciences, University of Barcelona, 08036 Barcelona, Spain; nicolas@ub.edu; 3 Departament of Neurosurgery, University Hospital Doctor Negrín, 35010 Las Palmas de Gran Canarias, Spain; david__carrera@hotmail.com; 4 Departament of Neurology, University Hospital Germans Trias i Pujol, 08916 Badalona, Spain; natperezossa@gmail.com

**Keywords:** stroke, prehospital emergency care, training, stroke code, large vessel occlusion, prehospital scales

## Abstract

Strokes are a time-dependent medical emergency. The training of emergency medical service (EMS) professionals is essential to ensure the activation of stroke codes with pre-notification, as well as a rapid transfer to achieve early therapy. New assessment scales for the detection of patients with suspected large vessel occlusion ensures earlier access to endovascular therapy. The aim of this study was to evaluate the impact on an online training intervention focused on the Rapid Arterial oCclusion Evaluation (RACE) scoring of EMS professionals based on the prehospital stroke code in Catalonia from 2014 to 2018 in a pre–post intervention study. All Catalonian EMS professionals and the clinical records from primary stroke patients were included. The Kirkpatrick model guided the evaluation of the intervention. Data were collected on the knowledge on stroke recognition and management, pre-notification compliance, activated stroke codes and time performance of EMS professionals. Knowledge improved significatively in most items and across all categories, reaching a global achievement of 82%. Pre-notification compliance also improved significantly and remained high in the long-term. Increasingly higher notification of RACE scores were recorded from 60% at baseline to 96.3% in 2018, and increased on-site clinical care time and global time were also observed. Therefore, the online training intervention was effective for increasing EMS professionals’ knowledge and pre-notification compliance upon stroke code activation, and the wide adoption of a new prehospital scale for the assessment of stroke severity (i.e., the RACE scale) was achieved.

## 1. Introduction

Strokes are a time-dependent medical emergency, in which treatment delay negatively influences patient prognosis [1]. For acute ischemic stroke patients with an evolution of less than 4.5 h of evolution, fibrinolysis therapy improves prognosis [2], yet its benefits are limited for the subgroup of patients with large vessel occlusion (LVO) [3]. These patients benefit the most from endovascular thrombectomy, with a therapeutic window of up to 24 h from stroke onset according to multimodal neuroimaging criteria, at an adequate specialized tertiary hospital, which doubles their chances of clinical improvement [4]. For this reason, in recent years, stroke code (SC) systems have been developed to rapidly identify patients with acute stroke, allowing agile transfers to a specialized center [5,6]. This rapid assessment of acute stroke patients is paramount to obtaining the maximum benefits from reperfusion therapies. Thus, emergency medical services (EMS) are essential [7], not only for identifying stroke patients, but also for identifying a subgroup of patients with suspected large vessel occlusion (LVO), who would benefit the most from endovascular treatment [8]. The traditional prehospital assessment scales were developed to detect the typical symptoms of stroke patients [9]. Since then, several new scales for the specific detection of LVO patients have been designed, but few have been validated prospectively in prehospital care [10]. Implementing these new prehospital diagnostic tools as part of SCs is a priority to ensure familiarity with the protocol and to achieve current therapeutic standards [11]. Giving pre-notification of patients to the receiving center is also important to ensure allocation of in-hospital resources and to accelerate diagnostic and therapeutic decision-making through a minimum set of clinical data [12]. Additionally, international guidelines also emphasized the need to prioritize specific training in SCs, diagnostic tools and pre-notification systems for EMS professionals [13,14,15]. Following these recommendations, our group developed the Rapid Arterial oCclusion Evaluation (RACE) [16] scale for prehospital assessment of patients with a suspected LVO stroke (Figure S1). The RACE scale was validated in 2014, and international guidelines endorsed the RACE scale as a valid tool alongside others [17,18]. For implementation by EMS professionals, an online training intervention (OTI) was designed to update their knowledge on acute stroke recognition and the SC activation circuit, as well as to train them on the administration of the RACE scale.

The aim of this study was to evaluate the impact on an OTI focused on the RACE scoring for EMS professionals based on prehospital SCs in Catalonia from 2014 to 2018.

## 2. Materials and Methods

We performed a pre–post intervention study from January 2014 to December 2018 in the Catalonian EMS (prehospital care). This EMS provides care for 7.5 million people, employing more than 4000 professionals, and it activated approximately eight daily SCs before 2014. For this study, we included data from both EMS professionals and stroke patients. All EMS professionals (i.e., emergency technicians, nurses, and physicians) were invited to participate, and all of those who accepted were included, as no exclusion criteria were considered. A non-probabilistic sampling method was used. All clinical records of patients older than 18 years old and classified as primary acute stroke patients upon activation of SCs by the dispatch center were included. Records of patients who were being transferred between hospital settings were excluded.

### 2.1. Online Training Intervention

An online training intervention (OTI) was developed to provide 6 h of training through a learning management system (i.e., Moodle). The programme comprised four modules: Three theoretical modules that addressed the (a) signs and symptoms of a stroke, (b) stroke treatment, and (c) prehospital management of stroke, including the administration of prehospital scales and SC protocol; the final module was practical, and was introduced to address the application of the RACE scale using five clinical scenarios. The contents and evaluation methods considered the recommendations of the Cerebrovascular Disease Master Plan in Catalonia to attain content validity. Additionally, international recommendations from the European and American Stroke Organizations were incorporated into the curriculum. A pilot test was performed with an interprofessional group of 30 individuals that included neurologists and EMS professionals (i.e., physicians, nurses, and emergency technicians) between March and April 2014. The training was accredited by the regional council for the continuous education of healthcare professionals.

The OTI was administered progressively according to the Catalonian healthcare regions. All professionals from the same region participated in the course simultaneously for a 30-day period with on-demand access to the training platform. Fourteen replications of the training program were necessary to cover all regions and professionals. The training was completed by 2830 EMS professionals from May to September 2014. All EMS professionals were supported by forum interactions with the faculty, and additional resources were provided. A collaboration network was established by developing a Facebook group, a Twitter account (@escalaRACE), and a website (www.racescale.org).

### 2.2. Assessment of the Online Training Programme

The variables measuring the effectiveness of the OTI with regard to prehospital SCs were categorized using Kirkpatrick’s [19,20] model of training evaluation as follows:

- Kirkpatrick level 1 (reaction): A satisfaction survey (Supplementary material S2) was administered at the end of the training using a five-point Likert scale based on five dimensions. Satisfaction of the aims of the training, the available materials, the RACE scale usefulness in clinical practice, and the faculty, as well as the overall satisfaction regarding the perceived increased in knowledge or competency, were addressed.
- Kirkpatrick level 2 (learning): A knowledge-related multiple-choice questionnaire about knowledge was administered prior to and 3 months after the intervention (Supplementary material S3). A set of 24 questions were used, most of which (i.e., 10) were related to the identification of signs and symptoms of a stroke, as well as available treatment options. Eight questions explored respondents’ knowledge on the SC protocol, prehospital management, and prehospital assessment scales for stroke patients. A final set of six questions were used to analyze the decision-making on acute stroke medical emergencies in clinical scenarios.
- Kirkpatrick level 3 (behavior): The transfer to clinical practice was measured by compliance rates with the EMS prenotification system (i.e., Minimum Data Set register). We observed the notification of the patient’s identification number, the time of the onset of symptoms, anticoagulant treatment, glycaemia, systolic and diastolic blood pressure, and RACE scores.
- Kirkpatrick level 4 (results): The impact of the intervention on prehospital SC was assessed by determining the number of activated codes and the changes in prehospital care times. The observed times were: (a) The alert time, that is, the period between the start and the end of a call; (b) the activation time, that is, the time from the start of a call to the allocation of clinical resources; (c) the response time, that is, the period from resource allocation to the arrival of the EMS team; (d) the care time, that is, the time from the arrival at the place of care to the start of the transfer; (e) the transfer time, that is, the period from the start of the transfer to the arrival at the receiving center; and (f) a global time was also registered.

### 2.3. Data Collection

This study was performed at five time-points: (a) Baseline (first quarter of 2014 (Q1)), (b) training intervention (second (Q2) and third quarter (Q3) of 2014), (c) immediate follow-up (fourth quarter of 2014 (Q4)), (d) follow-up after 1–2 years (2015–2016), and (e) follow-up after 3–4 years (2017–2018). The period between 2014 Q1 (baseline) and Q4 (immediate follow-up) was used to pilot the training intervention (March to April 2014); and to train the EMS professionals (May to September 2014).

Data from EMS professionals were obtained on the same learning management system (i.e., Moodle) on which the course was provided. Socio-demographics were obtained at the beginning of the intervention (i.e., 2014 Q2 and Q3). Data on Kirkpatrick level 1 was recorded at the end of the intervention (i.e., 2014 Q2 and Q3). Kirkpatrick level 2 data were obtained at the beginning of the intervention (i.e., 2014 Q2 and Q3), and 3 months after the end of the training (2014 Q4). Data on all results from Kirkpatrick levels 3 and 4 were obtained through the Informatic System for Emergency Management (SITREM^®^) register between 2014 and 2018 (all periods except the intervention, that is, 2014 Q2 and Q3). This register prospectively records information about all Catalonian EMS activity, including details on SC activation, patients, time of call, first time of care, and arrival at the receiving hospital. From September 2014, RACE scores were also included in the register.

The data were processed in compliance with the European Data Protection Regulation 2016/679. The study was approved by the Clinical Research Ethics Committee of the University Hospital Germans Trias i Pujol (Badalona, Spain) with identification code PI-15-030.

### 2.4. Statistical Analysis

The results are expressed in means and standard deviations (SDs) for quantitative variables, and absolute frequencies and percentages for qualitative variables.

The Student’s *t*-test was used for the comparative analysis of paired data. A *p*-value of <0.05 was considered statistically significant. Data were analyzed with the statistical software program SPSS version 24.0 (SPSS Inc.; Chicago, IL, USA).

## 3. Results

**Figure 1:**
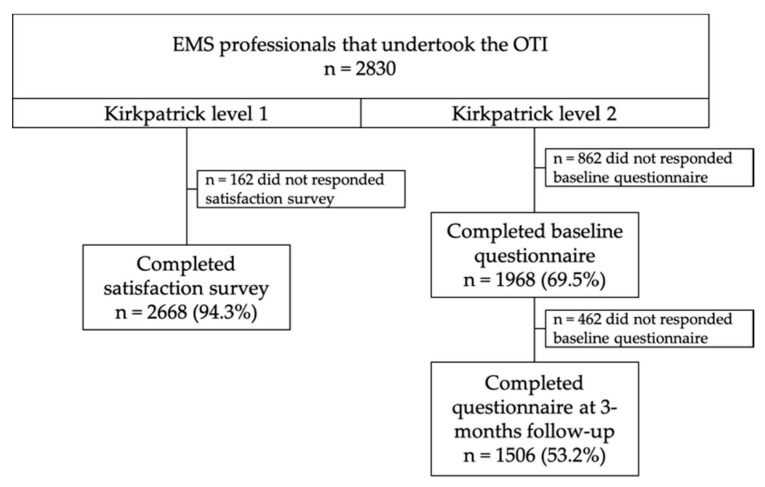
Flowchart of emergency medical services (EMS) professionals’ participation.

A total of 2830 EMS professionals undertook the training programme, and 69.5% completed the baseline questionnaire while 53.2% answered the three-month follow-up (Figure 1). The majority were males (76.7%) with a mean age of 35.8 years (SD = 6.3) and more than 10 years of experience in EMS (45.4%). Emergency medical technicians accounted for 90.3% of the staff, followed by nurses (6.4%) and then physicians (3.2%). Most of them worked as care providers (98.3%) and only 1.7% did so in the dispatch center. Meanwhile, 65% had received previous training on strokes. The satisfaction survey (Kirkpatrick level 1) at the end of the training was completed by 2668 (94.3%), scoring at least 4 out of 5 in all items.

### 3.1. Learning Assessment (Kirkpatrick Level 2)

After the OTI, there was a significant increase in 80% of the questions related to the recognition of the signs and symptoms of a stroke (Table 1), especially for those addressing location-specific signs, as well as the treatment. Two generic questions about strokes were non-significant but scored high prior to the training. The questions regarding SCs, prehospital management, and prehospital stroke assessment increased significantly, with most scores above 85%. Changes were observed in stroke recognition, with improved identification in 5/6 of the clinical scenarios.

### 3.2. Transfer to the Clinical Setting (Kirkpatrick Level 3)

The Minimum Data Set (MDS) records available in the SITREM^®^ register were analyzed for 17,135 patients in the study period (Table 2). Immediately after the training, we found a significant increase in the registration of patient identification (ID) codes, glycaemia, systolic and diastolic blood pressure (SBP/DBP), and RACE scores, yet notification of the time of the onset of symptoms decreased. For the 2015–2016 follow-up, only the patient ID and RACE records continued to increase, in contrast to the time of symptom onset (TSO), SBP and DBP. There were significant differences for all items in the 2017–2018 follow-up period: All items increased (i.e., TSO, glycaemia, ID, SBP, DBP, and RACE notification), and the frequency of anticoagulant treatment notification decreased. Overall, 71.5% of the items increased from baseline to the last follow-up. Only the registration of TSO and the notification of anticoagulant treatment diminished consistently over time.

Compliance with the RACE scale upon SC activation increased continuously over time. Starting from 60.9% immediately after training, compliance rose to over 85% by the 2017–2018 follow-up, reaching a 96.3% compliance level in 2018.

### 3.3. Impact on Prehospital Stroke Code (Kirkpatrick Level 4)

#### 3.3.1. Stroke Code Activation

Activation of SCs increased over time. At baseline (2014 Q1), 9.2 codes were activated daily (n = 834), which increased immediately after training (2014 Q4) to 10.7 (n = 965), maintaining at 9.9 in both 2015 (n = 2635) and 2016 (n = 3635). In 2017, 10.6 codes were initiated (n = 3888), which reached a daily maximum of 11.4 (n = 4187) in 2018.

#### 3.3.2. Time Performance in Stroke Code

The main differences in time performance were the overall time of prehospital care (Table 3), which increased from 48.9 to 53.6 min (*p* = 0.015). This extra time was mostly due to increased on-site clinical care time (*p* = 0.034) prior to transfer to hospital, which increased from 21.5 to 24.3 min. However, there were no changes in activation, alert, response, or transfer times.

## 4. Discussion

Time is brain when dealing with acute stroke patients. International Stroke Organizations advocate for the development of specific training programs for healthcare professionals, as those who have not been trained specifically on strokes contribute to delay patients’ access to adequate therapy [21]. On the other hand, the Stroke Alliance for Europe advocates for a systematic approach to continuous education in EMS as one of their 12 quality care indicators on strokes [22].

In this study, the OTI was well-received, scoring high in satisfaction (Kirkpatrick level 1). It was associated with a knowledge gain for all categories (Kirkpatrick level 2), as observed in similar studies with EMS professionals [23,24,25]. Participants accurately identified the signs and symptoms of a stroke and became aware of the differences between hemorrhagic and ischemic strokes. On the other hand, very few improvements were observed in recognizing transient ischemic attacks. Despite most EMS professionals (65%) having had previous education on strokes, a lot of heterogeneity was found at baseline for the recognition of very specific signs and symptoms. This training improved their competency, which is consistent with the improvements in knowledge observed by Hsieh et al. [23] in Taiwan, where 48% had previously received training. The window for thrombolytic therapy was only identified by a third of the participants, and half signaled the indications for endovascular treatment. However, these items might be very specific, which could explain the low number of correct answers in our case, or why a similar study in Dubai [24], received no correct responses for these questions. It should be noted that while diagnostic and comorbidity scales determine the activation of the SCs, only 50.3% and 40.1%, respectively, were familiarized with them, which is similar to the finding of DiBiasio in Rhode Island [25]. An integrative review [26] on the impact of training programs on strokes found that only 1 of 21 courses was taught online. In that single UK-based study (RESPONSE [27]), there was a greater improvement in knowledge compared to our findings (95.6% vs. 82%), while the response rates varied (39% vs. 54%). These differences could be explained by the fact that our study managed to include a greater percentage of EMS professionals and that the context of the education was different. Most professionals in RESPONSE were paramedics (55%), who had received 2–5 years of university training (Paramedic Sciences), in contrast to our 90.3% participation of emergency technicians, who had received a 2-year non-university qualification. This could also explain some of the very low scores at baseline when recognizing specific stroke signs and symptoms.

Improving pre-notification systems in SCs is essential for ensuring the allocation of in-hospital resources and for accelerating communication between EMS teams and receiving hospitals [28]. We observed a progressive improvement in the compliance with the pre-notification register in 80% of the items, increasing from an overall 53% compliance score in 2014 to a 73% in 2015–2016, and 83% in 2017–2018 (Kirkpatrick level 3). This increment was greater than in another study performed in the USA [29] that achieved an increase from 60.9% to 77.3%; the last peak of 10% increase in our case could have been due to the start of RACECAT, a clinical trial focused on different transfer approaches for stroke patients. Pre-notifications systems have also been found effective in improving in-hospital times for therapy access [30,31]. We observed fewer notifications of TSO, which could be explained by the presence of more cases of awakening strokes in the last period (2017–2018).

New specific scales for LVO patients have been created, but most are still uncommon in EMS [32,33,34]. Our group developed and validated the RACE scale [12] in 2014, which has received endorsement by international guidelines [35,36,37]. We documented a great compliance with the prehospital assessment of LVO patients with the RACE scale, starting at 61% immediately after training (Kirkpatrick level 3), up to 71% at the 1- to 2-year follow-up, and 91% in the 3- to 4-year follow-up. During the last year (i.e., 2018) compliance reached 96.3%, which is similar to that found in a study in Ohio (USA) [38] that reached 100% compliance in recoding RACE scores. The results of studies using other scales for LVO recognition are varied; for example, an Australian study [39] reached 88% notification, while another study involving multiple EMS agencies involved only provided data in 53% of the cases. In our study, support from the EMS directorate and continuous education department, as well as the inclusion of the RACE scores in the EMS clinical register (i.e., SITREM^®^), were paramount to achieving these positive long-term results.

Acute strokes are a time-dependent medical emergency where time between the onset of symptoms and treatment is essential. We recorded changes in prehospital care time as overall prehospital care time, which increased by 4.7 min (Kirkpatrick level 4). Additionally, on-site clinical care time increased by 2.8 min. A UK study, PASTA [40], showed an increase in time from assessment to thrombolysis by 8.5 min after a specific training programme for paramedics. Another UK project [41] focused on training at the dispatch center revealed a non-significant 2.8 min reduction in the time between alert activation to the arrival of the ambulance. The benefits of patient assessment using the RACE and the obtained pre-registration data (i.e., vital signs, assessment of stroke severity, and RACE scores) could outweigh the slight increase in the overall prehospital care time.

However, this study has some limitations. First, it was limited to the prehospital setting. Second, the correlation between the RACE score at prehospital assessment and endovascular therapy effectiveness remains unknown. Finally, we have no data on the prognosis and evolution of the stroke patients (i.e., final diagnosis, false positives or negatives, stroke mimics, and reperfusion therapy rates). Future studies should seek to include further in-hospital clinical variables.

## 5. Conclusions

An interprofessional OTI on strokes in the Catalonian EMS was effective in increasing the participants’ knowledge on cerebrovascular medical emergencies. Both strengths and areas for improvement were detected for future training opportunities. This study had a positive long-term impact on prehospital compliance with the pre-notification system upon SC activation. This training intervention permitted the wide adoption of a new prehospital scale for the assessment of stroke severity (i.e., the RACE scale), reaching high notification compliance.

These results encouraged the Catalonian EMS to maintain this training intervention in their continuous education program, which, starting back in 2015, is delivered twice a year.

## Figures and Tables

**Table 1:** Differences in the responses to the knowledge multiple-choice test.

Topics	Baseline (n = 1968)	After Training (n = 1506)	*p*-Value
	(% Correct)	(% Correct)	(* <0.05)
Signs and symptoms of a stroke	1960 (99.6)	1500 (99.7)	0.97
Ischemic stroke etiology	1934 (98.3)	1483 (98.5)	0.645
Hemorrhagic stroke etiology	1891 (96.1)	1491 (99.1)	<0.001 *
Left hemisphere stroke signs/symptoms	1029 (5.2)	925 (61.4)	<0.001 *
Right hemispheres stroke signs/symptoms	267 (13.7)	615 (40.9)	<0.001 *
Transient ischemic attack definition	1272 (64.6)	1024 (68.0)	0.038 *
Brainstem stroke signs/symptoms	1245 (63.2)	1240 (82.3)	<0.001 *
Window for thrombolysis	689 (35.0)	774 (51.4)	<0.001 *
Endovascular indications	974 (49.5)	1022 (67.9)	<0.001 *
Benefits of stroke treatment	1027 (52.2)	1209 (80.3)	<0.001 *
Aim of the stroke code	1833 (93.1)	1446 (96.0)	<0.001 *
Target age for the stroke code	1510 (76.7)	1430 (95.0)	<0.001 *
Time criteria from onset to code activation	1247 (63.3)	1322 (87.8)	<0.001 *
Criteria for activation of the stoke code	1251 (63.6)	1341 (89.1)	<0.001 *
Recognition of comorbidity scales	403 (40.1)	906 (60.2)	<0.001 *
Recognition of stroke diagnostic scales	1186 (50.3)	1393 (92.5)	<0.001 *
RAPID ^1^ scale recognition	1033 (52.5)	1236 (82.1)	<0.001 *
RANCOM ^2^ scale recognition	1616 (82.1)	1390 (92.3)	<0.001 *
Hypertension and nausea	1298 (65.9)	961 (63.8)	0.189
Transient ischemic attack	1534 (77.9)	1277 (84.8)	<0.001 *
90 years. 5 h start of symptoms	1515 (77.0)	1364 (90.6)	<0.001 *
Treatment with anticoagulants	1560 (79.3)	1288 (85.5)	<0.001 *
Awakening stroke	1644 (83.5)	1430 (95.0)	<0.001 *
RANCOM ^2^ scale	1309 (66.5)	1145 (76.0)	<0.001 *

^1^ The smile, raise the arm, talk, stroke, call 911 fast mnemotechnic (i.e., RAPID) is a Catalan stroke assessment tool, equivalent to the FAST mnemotechnic in English-speaking countries. ^2^ Rankin Comorbidity (i.e., RANCOM) is a Catalan comorbidity scale for prehospital assessment of stroke patients. * *p* < 0.05.

**Table 2:** Differences in the pre-notification items from the Minimum Data Set register over time.

Item	Baseline (2014)	Immediately after Training (2014 Q4)		Immediately after Training (2014 Q4)	1–2 Years after Training (2015–2016)		1–2 Years after Training (2015–2016)	3–4 Years after Training (2017–2018)	
	n = 834	n = 965		n = 965	n = 7261		n = 7261	n = 8075	
	n (%)	n (%)	*p*-Value	n (%)	n (%)	*p*-Value	n (%)	n (%)	*p*-Value
Patient identification no.	460 (55.2%)	632 (65.5%)	<0.001 *	632 (65.5%)	4998 (68.8%)	0.036 *	4998 (68.8%)	6904 (85.5%)	<0.001 *
Time from onset of symptoms	714 (85.6%)	759 (78.7%)	<0.001 *	759 (78.7%)	5305 (73.1%)	<0.001 *	5305 (73.1%)	6211 (76.9%)	<0.001 *
Anticoagulant therapy	704 (84.4%)	809 (83.8%)	0.738	809 (83.8%)	6237 (85.9%)	0.086	6237 (85.9%)	6327 (78.3%)	<0.001 *
Glycaemia	246 (29.5%)	672 (69.6%)	<0.001 *	672 (69.6%)	4922 (67.8%)	0.247	4922 (67.8%)	6524 (80.8%)	<0.001 *
Systolic blood pressure	255 (30.6%)	711 (73.7%)	<0.00 1*	711 (73.7%)	5043 (69.5%)	0.009 *	5043 (69.5%)	7007 (86.8%)	<0.001 *
Diastolic blood pressure	254 (30.5%)	711 (73.7%)	<0.001 *	711 (73.7%)	5044 (69.5%)	0.007 *	5044 (69.5%)	6516 (80.7%)	<0.001 *
RACE scores	---------------	588 (60.9%)		588 (60.9%)	5165 (71.1%)	<0.001 *	5165 (71.1%)	7350 (91.0)	<0.001 *

RACE, Rapid Arterial oCclusion Evaluation; * *p* < 0.05.

**Table 3:** Differences in time performance over time.

Item	Baseline (2014 Q1) n = 834	Immediately after Training (2014 Q4) n = 965	1–2 Years after Training (2015–2016) n = 7261	3–4 Years after Training (2017–2018) n = 8075	95%CI of the Difference	*p*-Value
	Mean (SD)	Mean (SD)	Mean (SD)	Mean (SD)		
Alert time ^1^	3.10 (6.7)	2.96 (5.6)	2.90 (5.8)	3.13 (6.1)	−0.25 to 0.56	0.877
Activation time ^2^	4.78 (7.1)	4.97 (6.7)	4.86 (7.0)	5.35 (7.6)	−1.64 to 0.73	0.053
Response time ^3^	8.08 (4.9)	8.68 (5.3)	8.57 (5.6)	8.70 (6.0)	−0.34 to 1.23	0.083
Clinical care time ^4^	21.51 (8.4)	21.87 (8.3)	22.24 (8.9)	24.32 (8.9)	0.75 to 3.27	0.034 *
Transfer time ^5^	12.29 (10.1)	13.11 (10.6)	12.73 (10.3)	12.86 (11.2)	−1.51 to 1.66	0.402
Overall time ^6^	48.9 (19.9)	51.60 (19.5)	51.52 (19.6)	53.62 (20.0)	1.04 to 5.33	0.015 *

^1^ Alert time: The time between the start and the end of a call at the dispatch center. ^2^ Activation time: The time from the start of a call to the allocation of clinical resources at the dispatch center. ^3^ Response time: The period from the resource allocation to the arrival of the EMS team at point of care. ^4^ Clinical care time: The time from the arrival of the EMS team at the point of care to the start of transfer; on-site care is provided. ^5^ Transfer time: The time of transportation from the point of care to arrival at the receiving center. ^6^ Overall time: The sum of all previous times. CI, confidence interval; SD standard deviation. * *p* < 0.05.

## Supplementary Materials

The following are available online at https://www.mdpi.com/1660-4601/17/17/6183/s1, Figure S1: Summary table RACE Scale and Instructions to evaluate the RACE scale, S2: satisfaction survey, S3: knowledge-related multiple-choice questionnaire.
