# Supplementary material for: An Online Training Intervention on Prehospital Stroke Codes in Catalonia to Improve the Knowledge, Pre-Notification Compliance and Time Performance of Emergency Medical Services Professionals
Source: Int J Environ Res Public Health. 2020 Aug 26;17(17):6183. doi: 10.3390/ijerph17176183 (PMC7503298; doi:10.3390/ijerph17176183)
Supplement: Supplementary file 1 [file ijerph-17-06183-s001.zip › supplementary/RACE scale.pdf]

RACE SCALE

Germans Trias i Pujol Hospital
Institut Català de la Salut

emergències mèdiques

RACE SCALE	
**1. Facial palsy:**	
Facial movement is normal, symmetric	0
Facial gesture when showing the teeth or smiling is slightly asymmetrical	1
Facial gesture when showing the teeth or smiling is completely asymmetrical	2
**2. Arm motor function:**	
Maintain the arm against gravity >10 seconds	0
Maintain the arm against gravity <10 seconds	1
Cannot maintain the arm against gravity and drops immediately	2
**3. Leg motor function:**	
Maintain the leg against gravity >5 seconds	0
Maintain the leg against gravity <5seconds	1
Cannot maintain the leg against gravity and drops immediately	2
**4. Head and gaze deviation**	
Absent	0
Present	1
**5A. Agnosia / Negligence (if left hemiparesis)**	
Asomatognosia (do not recognize the left part of his/her body)<br>*Anosognosia (do not recognize his/her weakness)*	
There is no asomatognosia nor anosognosia	0
There is asomatognosia or anosognosia	1
There is asomatognosia and anosognosia	2
**5B. Aphasia / Language (if right hemiparesis)**	
Ask the patient: “Close your eyes” and “Make a fist”	
Perform both tasks correctly	0
Perform one task correclty	1
Perform neither tasks	2
**TOTAL**	
