# Supplementary material for: An Online Training Intervention on Prehospital Stroke Codes in Catalonia to Improve the Knowledge, Pre-Notification Compliance and Time Performance of Emergency Medical Services Professionals
Source: Int J Environ Res Public Health. 2020 Aug 26;17(17):6183. doi: 10.3390/ijerph17176183 (PMC7503298; doi:10.3390/ijerph17176183)
Supplement: Supplementary file 1 [file ijerph-17-06183-s001.zip › supplementary/RACE scale instructions.pdf]

Instructions to evaluate the RACE scale

The RACE scale evaluates 5 items. The last item is different for patients with right or left hemiparesis.

Facial palsy

Ask the patient to smile or to show his/her teeth. Score the symmetry of the facial movement. If the patient does not understand or can not collaborate, application of pressure with the fingers behind the ears can provoke facial grimacing.

0: Facial movement is normal, symmetric.
1: Facial gesture when showing the teeth or smiling is slightly asymmetrical
2: Facial gesture when showing the teeth or smiling is completely asymmetrical

Arm motor function

To evaluate for arm hemiparesis, ask the patient to raise the arms, 45 degrees if the patient is in supine position or 90 degrees if he/she is sitting. If the patient does not collaborate, extend his/her arms up. Score according to the time the patient can maintain his arms against gravity, without touching the bed or the surface.

0: The patient can maintain the arm against gravity up to 10 seconds
1: The patient can maintain the arm less than 10 seconds
2: The patient cannot maintain the arm against gravity and drops immediately.

Leg motor function

To evaluate for leg hemiparesis, ask the patient to raise each leg 30 degrees with the patient in supine position. If the patient does not collaborate, lift the legs up. Score according to the time the patient can maintain the leg against gravity, without touching the bed or the board where he is sitting.

0: The patient can maintain the leg against gravity up to 5 seconds.
1: The patient can maintain the leg less than 5 seconds
2: The patient cannot maintain the leg against gravity and the leg drops immediately

Head and gaze deviation

Observe eyes and head deviation to one side.

0: Absent
1: Present

Agnosia / Negligence (if left hemiparesis)

1. Evaluate if the patient recognizes his/her left part of the body: Ask the patient “Whose arm is this?” while showing him/her the paretic arm. Patients with asomatognosia do not recognize the left part of their body.
2. Evaluate if the patient is aware of the weakness: Ask the patient: “Can you move your arms and clap your hands?”. Patients with anosognosia do not recognize their weakness.

0: There is no asomatognosia nor anosognosia
1: There is asomatognosia or anosognosia
2: There is asomatognosia and anosognosia

Aphasia / Language (if right hemiparesis)

Ask the patient two verbal orders: “Close your eyes” and “Make a fist” (always use these commands)

0: Performs both tasks correctly
1: Performs one task correctly
2: Performs neither task
