# Supplementary material for: An Online Training Intervention on Prehospital Stroke Codes in Catalonia to Improve the Knowledge, Pre-Notification Compliance and Time Performance of Emergency Medical Services Professionals
Source: Int J Environ Res Public Health. 2020 Aug 26;17(17):6183. doi: 10.3390/ijerph17176183 (PMC7503298; doi:10.3390/ijerph17176183)
Supplement: Supplementary file 1 [file ijerph-17-06183-s001.zip › supplementary/Satisfaction survey.pdf]

SATISFACTION SURVEY

The aim of the survey is to evaluate the relevance of the course on acute stroke and selection of patients for endovascular treatment with the RACE scale. The answers are anonymous, and the results will be observed aggregately.

Age ____________

Gender:

□ Female □ Male □ Other

Indicate the healthcare region you are working in:

- □ Barcelona Ciutat
- □ Barcelonès Sud
- □ Barcelonès Nord
- □ Camp de Tarragona i Terres de l'Ebre
- □ Catalunya Central
- □ Girona
- □ Lleida, Alt Pirineu, Aràn

State your Profession

- □ Emergency technician
- □ Nurse
- □ Physician

Working experience in prehospital care

- □ Less than 5
- □ 6 to 10
- □ 10 years or more

Area of professional work

- □ Clinical care
- □ Dispatch center

How do you rate your knowledge about stroke?

- □ Very poor
- □ Poor
- □ Regular
- □ Good
- □ Very good
- □ Expert

Had you previously received specific training on the Stroke Code and its activation criteria?

- □ Yes, in a particular occasion
- □ Yes, sporadically
- □ No
- □ NS/NC

SATISFACTION SURVEY

For the following question, choose the option that you consider more accurate according to your degree of satisfaction.

	Completely disagree	Disagree	Not agree nor disagree	Agree	Completely agree
	1	2	3	4	5
1.- Program goals					
The aims of this training program					
2.- Course materials					
2.1. Organization of the virtual classroom					
2.2. Rate the theoretical contents					
2.3. Rate the practical contents					
2.4. Quality of additional materials					
2.5. Technical functioning of the classroom					
3.- RACE scale usefulness in clinical practice					
3.1 RACE scale – Is it easy to use?					
3.2 RACE scale – Is it useful in clinical practice?					
4.- Faculty					
4.1 Proficiency level of the faculty staff					
4.2 Ability to solve doubts/questions					
5.- Overall satisfaction					
5.1 The course has helped you to improve your knowledge of cerebral vascular pathology					
5.2 The course has met your training expectations					
