# Supplementary material for: An Online Training Intervention on Prehospital Stroke Codes in Catalonia to Improve the Knowledge, Pre-Notification Compliance and Time Performance of Emergency Medical Services Professionals
Source: Int J Environ Res Public Health. 2020 Aug 26;17(17):6183. doi: 10.3390/ijerph17176183 (PMC7503298; doi:10.3390/ijerph17176183)
Supplement: Supplementary file 1 [file ijerph-17-06183-s001.zip › supplementary/Knowledge test.pdf]

KNOWLEDGE QUESTIONNAIRE

1. What is the main goal of the activation of the stroke code (SC)?

- The administration of thrombolytic treatment
- The admission to a stroke unit
- To identify and accelerate the initial diagnosis of stroke, so the appropriate therapeutic decisions can be made as quickly as possible
- N/a

2. How do you assess the patient's age when alerting the coordination center of the possible activation of the stroke code?

- I activate the SC if the patient is 70 or younger
- I activate the SC if the patient is 80 or younger
- I activate the SC if the patient is 85 or younger
- There is no age limit in the activation of the stroke code

3. How do you assess the stroke evolution time when alerting the coordination center of the possible activation of the stroke code?

- I activate the SC if the stroke evolution time is less than 3 hours
- I activate the SC if the stroke evolution time is less than 4,5 hours
- I activate the SC if the stroke evolution time is less than 8 hours or it's a stroke of uncertain chronology, or upon awakening
- I activate the SC regardless of the evolution time, provided it was less than 24 hours
- N/a

4. Are you familiar with any scale to assess the comorbidity or functional medical condition of the patient with suspected acute stroke?

- No, I don't use any scale
- I ask the relatives about the patient's prior quality of life
- I use the RANCOM scale
- I use the RANKIN scale

5. Which of the following hints and/or symptoms would make you suspect a stroke?

- A sudden headache
- Difficulty in speech, weakness on one side of the body, mouth deviation to one side
- Loss of consciousness

6. Do you currently use a scale for stroke diagnosing?

- Yes, the Cincinnati scale
- Yes, the fast scale
- Yes, RAPID scale
- No, just physical exam
- N/a

7. Ischemic stroke is due to the occlusion of a large cerebral vessel.

- True
- False

8. Hemorrhagic stroke is cause of a cerebral circulation artery breakdown:

- True
- False

9. A woman calls the EMS (Emergency Medical Services) because she doesn't understand her husband when he speaks. Upon arrival of the EMS team to their home, the patient is not able to speak, nor he understands what is said (he suffers aphasia), and he does not move his right limbs. He has an 220/105 arterial blood pressure and has nausea.

- He suffered an ischemic stroke due to a small blood vessel occlusion of the cerebral circulation
- He suffered an ischemic stroke due to a great blood vessel occlusion of the cerebral circulation
- He suffered a hemorrhagic stroke due to his high blood pressure (hypertension) and has nausea
- You suspect that he had a stroke, but cannot determine whether it was an ischemic or a hemorrhagic stroke
- N/a

10. A patient with a stroke that affects his/her left hemisphere will not present:

- Deviation of the gaze leftwards
- Weakness of the left side limbs
- Aphasia
- Hemianopsia (loss of part of his visual field) on his right side
- N/a

11. A patient with a stroke that affects his right hemisphere will not present:

- Deviation of the gaze rightwards
- Weakness of the left side limbs
- Aphasia
- Hemianopsia (loss of part of his visual field) on his left side
- N/a

12. A transient ischemic attack is a neurological deficit due to the transitory occlusion of a cerebral circulation artery. Mark the correct answer:

- Symptoms are mild, they last a few days and they don't affect the patient's functional situation
- Symptoms disappear in less than 24 h
- There is no trace of injury to the brain's tissue
- Answers 1 and 2 are both correct

13. The RAPID tool helps identify patients with acute stroke. We must ask the patient to do three different actions. Mark the incorrect answer:

- Laugh
- Raise his/her arms
- Shake hands
- Talk

14. A patient calls the EMS (Emergency Medical Services) because he/she feels a sudden weakness on his/her left-side limbs which started half an hour ago. Upon arrival to his/her home, he/she lacks symptoms and you suspect he/she suffered a transient ischemic attack

- Patient must be taken to a hospital after activation of the stroke code
- Patient must be taken to a hospital where the cerebral vascular study can be performed in 24 hours (a hospital with a stroke unit)
- You must recommend the patient to visit his general practitioner
- You must recommend the patient to take an aspirin

15. A patient with a cerebral vessel stroke may present:

- Aphasia
- Double vision, dizziness, instability when walking, decreased level of consciousness
- Intense headache
- None of the above
- N/a

16. The RANCOM tool helps to assess the previous functional situation of acute stroke patients. The patient or his relatives must be questioned about three different areas. Mark the incorrect answer

- Mobility
- Use of bathroom
- Getting dressed
- Grocery shopping

17. To activate the Stroke code, the patient has to meet three criteria. Mark the incorrect answer:

- Aged under 80
- Evolution time less than 8 hours or unknown chronology
- Rapid +
- Negative RANCOM

18. A 90-year-old patient felt a weakness in his/her left limbs for 5 hours. The patient lives alone and does not need help to carry out his/her daily activities.

- Yes. I'd definitely activate the stroke code
- No. I wouldn't activate the stroke code

19. The cashier of a supermarket calls the Medical Emergency Services because one of her patrons just fell down the floor. When assessing the patient, he/she cannot speak and does not move his/her right limbs. Among his/her belongings you find some sintrom tablets.

- Yes, I'd definitely activate the stroke code
- No, I wouldn't activate the stroke code

20. 74-year-old sick person, who previously had a good quality of life; he/she went to bed the night before at 11.30pm feeling all right. His/her family found him/her in bed at 7:30am, unable to speak and feeling weak on the right side of his/her body.

- Yes, I'd definitely activate the stroke code
- No, I wouldn't activate the stroke code

21. A 60-year-old patient has been feeling weakness in his right limbs for the last hour. A week ago, he/she had surgery on his/her knee, and she/he walks with a crutch and needs help to get dressed ant to shower, but it's a temporary deficit

- Yes, I'd definitely activate the stroke code
- No, I wouldn't activate the stroke code

22. Intravenous fibrinolysis is the only revascularization treatment which has proven to be beneficial to patients with acute ischemic stroke. However, before administering it, we must check that the evolution time was....

- Less than 8 hours
- Less than 6 hours
- Less than 4,5 hours
- Less than 3 hours
- N/a

23. Mechanical thrombectomy is a revascularization treatment that allows to extract the thrombus that obstructs the cerebral artery by means of a selective catheterization. This treatment is useful...

- In all strokes
- In all ischemic strokes
- In some ischemic strokes due to large cerebral vessel occlusion
- In some ischemic strokes due to small cerebral vessel occlusion
- N/a

24. What pharmacological treatment has shown a clinical benefit to patients with acute stroke?

- Aspirin ®
- Sintrom®
- Thrombolysis with rtPa
- There is no treatment
- N/a
